# A 12-b Subranging SAR ADC Using Detect-and-Skip Switching and Mismatch Calibration for Biopotential Sensing Applications

**DOI:** 10.3390/s22093600

**Published:** 2022-05-09

**Authors:** Cong Luong Nguyen, Huu Nhan Phan, Jong-Wook Lee

**Affiliations:** Information and Communication System-on-Chip (SoC) Research Center, School of Electronics and Information, Kyung Hee University, Yongin 17104, Korea; luongnguyen@savarti.com (C.L.N.); phnhan2310@gmail.com (H.N.P.)

**Keywords:** analog-to-digital converter, successive approximation register, signal-to-noise, merged capacitor switching, capacitor mismatch

## Abstract

This paper presents a 12-b successive approximation register (SAR) analog-to-digital converter (ADC) for biopotential sensing applications. To reduce the digital-to-analog converter (DAC) switching energy of the high-resolution ADC, we combine merged-capacitor-switching (MCS) and detect-and-skip (DAS) methods, successfully embedded in the subranging structure. The proposed method saves 96.7% of switching energy compared to the conventional method. Without an extra burden on the realization of the calibration circuit, we achieve mismatch calibration by reusing the on-chip DAC. The mismatch data are processed in the digital domain to compensate for the nonlinearity caused by the DAC mismatch. The ADC is realized using a 0.18 μm CMOS process with a core area of 0.7 mm^2^. At the sampling rate *f*_S_ = 9 kS/s, the ADC achieves a signal-to-noise ratio and distortion (SINAD) of 67.4 dB. The proposed calibration technique improves the spurious-free dynamic range (SFDR) by 7.2 dB, resulting in 73.5 dB. At an increased *f*_S_ = 200 kS/s, the ADC achieves a SINAD of 65.9 dB and an SFDR of 68.8 dB with a figure-of-merit (FoM) of 13.2 fJ/conversion-step.

## 1. Introduction

Portable biomedical sensing applications demand low-power consumption for long battery operation. The human biopotentials have low-frequency bandwidth, up to a few kHz [1]. The amplitude of an electrocardiogram (ECG) is around 1 mV. An electroencephalogram (EEG) has an amplitude from 10 to 100 µV over a frequency band from 0.5 Hz to 150 Hz. The local field potential (LFP) has a typical amplitude of 1 mV over 1 Hz to 200 Hz. The biopotentials are low-amplitude signals, which must be amplified before signal processing. The next important block for signal processing will be the analog-to-digital converter (ADC). Thus, the performance of the amplifier and ADC determines the quality of the measured biopotentials. For digitizing the amplified signal, successive approximation register (SAR) ADC is suitable, with its energy-efficient structure for medium resolution. Moreover, the scaling-friendly structure of the SAR ADC has drawn continued research interest [2]. The basic building blocks of the SAR ADC include a comparator, a digital-to-analog converter (DAC), and SAR logic. The power consumption of the SAR logic, which is mostly digital, can be reduced by lowering the supply voltage. The comparator power can be reduced using a dynamic structure. Thus, researchers have investigated various energy-efficient DAC switching methods—for example, split-DAC [3], monotonic switching [4], set and down [5], and energy saving [6]. The work in [7] introduces a merged-capacitor-switching (MCS) method. In this approach, DAC capacitors are switched from the common-mode (CM) voltage *V*_CM_ to ground or reference voltage *V*_REF_. This method not only saves switching energy but also effectively handles the issues related to CM variations. Behavioral simulation of a 10-bit SAR ADC shows 93.4% less switching energy than the conventional method.

A high-resolution SAR ADC demands a relatively large DAC area and switching energy. Switching energy is usually dominant in the capacitors of the most significant bit (MSB) segment rather than those in the least significant bit (LSB) segment. The work in [8] achieves efficient DAC switching using detect-and-skip (DAS). This approach allows skipping capacitor switching in the MSB segment, still generating the correct residue for the DAC. Implemented in 40 nm CMOS, the 10-bit SAR ADC achieves a signal-to-noise ratio and distortion (SINAD) of 55.6 dB and a spurious-free dynamic range (SFDR) of 76.2 dB; however, this work uses a split capacitor for the DAC, which is suboptimal in terms of energy efficiency compared to the MCS. If we can combine the merits of DAS and MCS, this approach can further reduce the DAC switching energy. Moreover, the work in [8] does not support DAC calibration; therefore, it cannot handle the mismatch caused by the parasitics and process variations.

Several approaches investigate techniques for DAC mismatch calibration. The work in [9] presents an on-chip dual calibration method for comparator offset and DAC mismatch. The work in [10] presents an energy-efficient ADC using digital domain calibration without additional analog circuits. The work in [11] presents a 13-bit SAR ADC with on-chip calibration for capacitor error compensation. Implemented in 130 nm CMOS, the ADC achieves a SINAD of 66.3 dB and an SFDR of 71 dB by consuming 1.47 μW; this approach suffers from a relatively large area of 0.9 mm^2^. A digital calibration method is presented using a sub-radix-2 redundant architecture [12], which can handle dynamic errors in the conversion process. The work in [13] analyzes the characteristics of the nonbinary-weighted capacitive DAC and a bottom-up weight calibration technique; however, these works have the drawback of altering the full-scale weight, which is different from the ideal value.

In this work, we present a 12-bit subranging SAR ADC suitable for low-power biopotential sensing applications. We reduce the DAC switching energy by combining MCS and DAS methods, successfully embedded in the subranging structure. Behavioral simulation of a 12-bit SAR ADC shows that the proposed method reduces 96.7% of switching energy compared to the conventional method, which is up to 9.2% lower than the previous state-of-the-art [7]. Without an extra burden on the realization of the on-chip calibration circuit, we implement digital domain calibration to compensate for the nonlinearity caused by the DAC mismatch. To address the drawback of the previous approach altering the weight of the full scale, we adopt a normalized full-scale weight for the subranging ADC. Using the proposed calibration technique, the ADC fabricated in 0.18 μm CMOS demonstrates successful operation and performance improvement. At a sampling rate of 200 kS/s, the ADC achieves a SINAD of 65.9 dB with a figure-of-merit (FoM) of 13.2 fJ/conversion-step. An SFDR of 68.8 dB is achieved near the Nyquist frequency. The novelty of this work is efficiently combining MCS and DAS for a high-resolution ADC and implementing a digital domain calibration using a normalized full-scale weight for the subranging ADC. 

## 2. Design

### 2.1. Subranging SAR ADC

**Figure 1:**
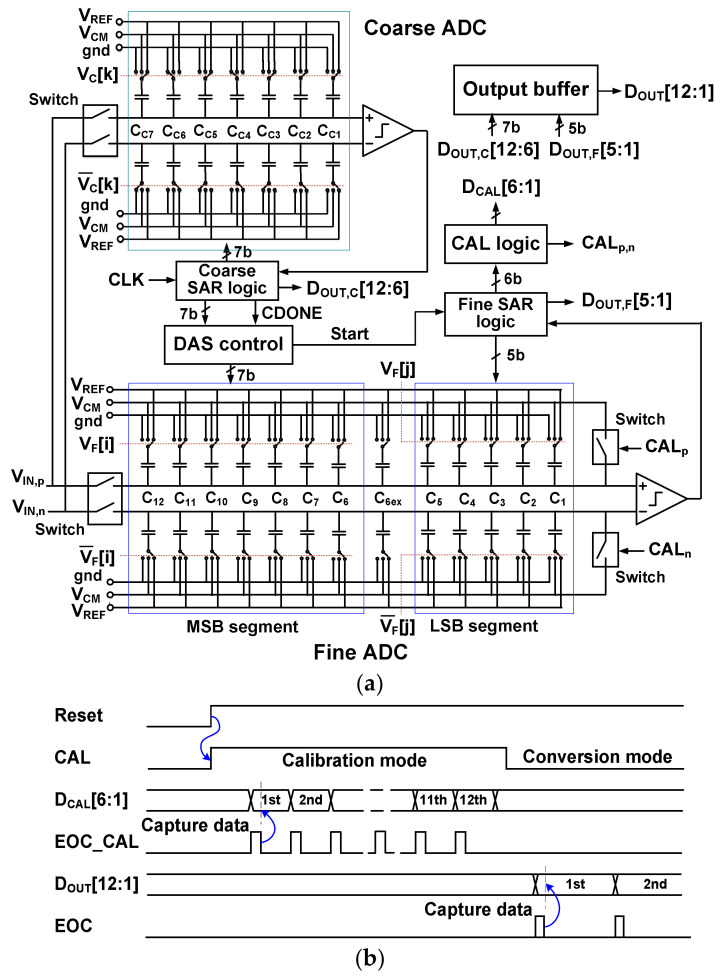
(**a**) Block diagram of the subranging ADC. (**b**) Timing sequence of the ADC.

Figure 1a shows the proposed subranging ADC. It includes a 7-bit coarse SAR ADC, a 12-bit fine SAR ADC, a DAS controller, a calibration (CAL) logic, and an output buffer. The coarse ADC includes the DAC consisting of seven binary-weighted capacitors *C*_Ck_ (*k* = 1 to 7). The fine ADC includes the DAC designed with twelve binary-weighted capacitors. For mismatch calibration, we divide the DAC into a 7-bit MSB segment of capacitors *C*_i_ (*i* = 6 to 12) and a 5-bit LSB segment of capacitor *C*_j_ (*j* = 1 to 5). MCS is used for coarse and fine ADCs to save DAC switching energy.

The analog input is sampled into the two ADCs at the same time. Top-plate sampling is performed using a bootstrapped switch operating with 1.8 V [9]. After sampling the input, the coarse ADC sequentially generates 7-bit output *D*_OUT,C_[12:6]. Then, the DAS controller and fine ADC are enabled by the signal CDONE (coarse done). The DAS controller decodes *D*_OUT,C_[12:6], and sets the switches for *C*_i_ (*i* = 6 to 12) of the fine ADC. This operation generates the residue in the fine DAC. Then, the SAR logic of the fine ADC sequentially determines the switch states of the remaining *C*_j_ (*j* = 1 to 5) to generate *D*_OUT,F_[5:1]. The *D*_OUT,C_[12:6] and *D*_OUT,F_[5:1] are combined in the output buffer to generate the ADC output *D*_OUT_[12:1] with the end-of-conversion (EOC) signal.

Figure 1b shows the timing sequence for the subranging ADC, which consists of calibration and conversion modes. The calibration mode includes three steps: reset, mismatch measurement, and data loading. When the reset signal becomes high, calibration mode starts with the calibration-enabled signal CAL. In this mode, the DAC inputs are disconnected from the analog input. During this time, the calibration code *D*_CAL_[6:1] for *C*_i_ (*i* = 6 to 12) is generated and loaded two times (positive and negative DAC). Two bootstrap switches are used to set the bottom plate of the DAC capacitor to *V*_CM_. These switches are controlled by the output CAL_p,n_ of the CAL logic at the beginning of each calibration cycle. The data loading occurs at the falling edge of EOC_CAL (end of calibration), which captures *D*_CAL_[6:1]. After finishing the calibration, the ADC enters conversion mode.

**Figure 2:**
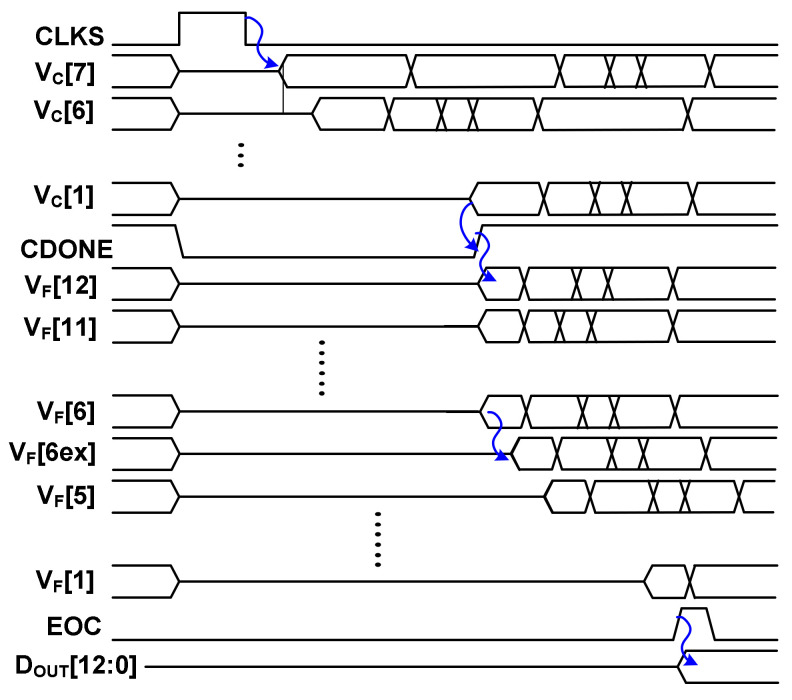
Timing sequence of the ADC in the conversion mode.

Figure 2 shows the timing sequence of the ADC in conversion mode. It shows the internal DAC control signals, *V*_C_[*k*] (*k* = 1 to 7) for the coarse ADC, *V*_F_[i] (*i* = 6 to 12) for the MSB segment of the fine ADC, and *V*_F_[j] (*j* = 1 to 5, 6ex) for the LSB segment of the fine ADC. *V*_F_[6ex] is the control signal for *C*_6ex_, which is an additional capacitor for mismatch calibration. The input signal is sampled into the coarse and fine ADCs by the sampling clock CLKS. All *V*_C_[*k*], *V*_F_[i], and *V*_F_[j] are connected to *V*_CM_. One cycle after CLKS, *V*_C_[*k*] is switched to either *V*_REF_ or ground, depending on the comparator output. After *V*_C_[1] is determined, the signal CDONE becomes high, indicating that the coarse ADC has finished quantization. Then, the DAS controller is enabled, which decodes *D*_OUT,C_[12:6] from the coarse ADC to determine *V*_F_[i] using the DAS operation. The controller decides which *V*_F_[i] is skipped or switched (either *V*_REF_ or ground) to generate the residue for *V*_DAC,p_ and *V*_DAC,n_ of the fine DAC. Here, *V*_DAC,p_ and *V*_DAC,n_ are the top-plate voltage of the positive and negative DAC, respectively. The fine ADC waits for one cycle after *V*_F_[i] switching so that the values of *V*_DAC,p_ and *V*_DAC,n_ are stabilized before it starts quantizing the remaining *V*_F_[j].

The input CM voltage is constant during MCS. In the previous work [4,9], the comparator is implemented with a PMOS differential pair because this comparator is designed for monotonic switching. When the previous comparator is used for MCS, it can result in a relatively large offset at the input of the comparator. In this work, we use a comparator having complementary input stages, which allows rail-to-rail range and reduces the kickback noise [14].

### 2.2. Merged Capacitor Switching with Detect and Skip

**Figure 3:**
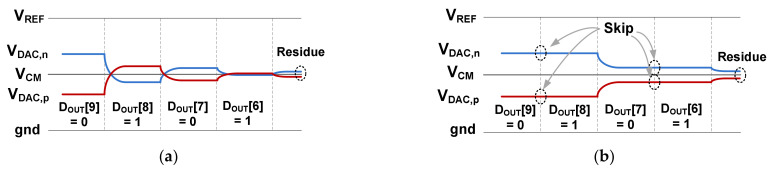
Example waveform of the DAC switching using (**a**) MCS only, (**b**) MCS and DAS method.

Figure 3a shows an example waveform of the DAC when *D*_OUT_[9:6] = 0101 is generated using MCS. Figure 3b shows the waveform when MCS and DAS are combined. The two methods generate the same residue for *V*_DAC,p_ and *V*_DAC,n_; however, MCS can waste energy by performing unnecessary switching. By combining MCS and DAS, unnecessary switching can be avoided. The DAS controller decides which capacitor can be skipped for switching. Using *D*_OUT,C_[12:6] from the coarse ADC, the DAS operation can be summarized as follows:

(1) *D*_OUT,C_[MSB-1] = *D*_OUT,C_[MSB] → switch *C*_MSB_|*D*_OUT,C_[MSB-1] ≠ *D*_OUT,C_[MSB] → skip *C*_MSB_,
(2) *D*_OUT,C_[MSB-2] = *D*_OUT,C_[MSB] → switch *C*_MSB-1_|*D*_OUT,C_[MSB-2] ≠ *D*_OUT,C_[MSB] → skip *C*_MSB-1_, …,
(3) *D*_OUT,C_[MSB-*k* + 1] = *D*_OUT,C_[MSB] → switch *C*_MSB-N+2_|*D*_OUT,C_[MSB-*k* + 1] ≠ *D*_OUT,C_[MSB] → skip *C*_MSB-N+2_,
(4) Switch *C*_MSB-N+1_,

Where MSB = 12 and *k* is the binary capacitor index of the coarse ADC. We note that the MCS and DAS method is more effective for a relatively smaller input since most switching can be skipped. Because the mismatch effect of the skipped capacitors is also removed, DAS can provide the additional benefit of improved linearity.

**Figure 4:**
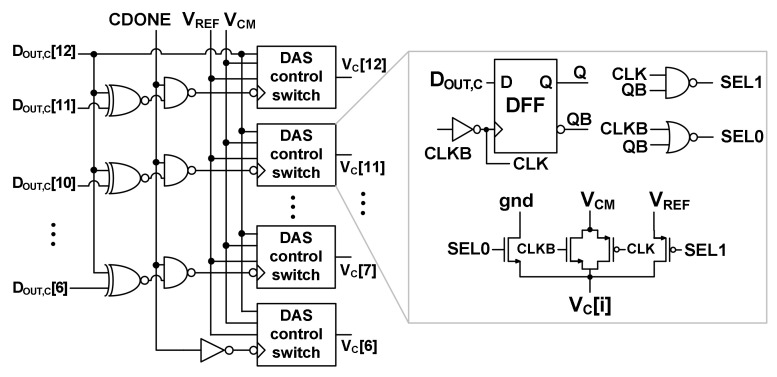
Schematic of the DAS controller.

Figure 4 shows the schematic of the DAS controller. When CDONE is enabled, the output *D*_OUT,C_[12:6] is input to the DAS control switch through the logic gates. In the beginning, *V*_C_[i] is connected to *V*_CM_. Depending on the logic value, *V*_C_[i] is connected to the ground if *D*_OUT,C_[i] is high, or *V*_C_[i] is connected to *V*_REF_. To evaluate the effectiveness of various switching methods, we compare the switching energy of a 12-bit ADC. The switching energy *E*_Mono_(i) of the *i*th capacitor in the monotonic switching can be expressed as

(1) $$E_{\mathrm{Mono}}(i)=\frac{C_{N-i+1}}{C_{T}}V_{\mathrm{REF}}^{2}\left[C_{T}-C_{N-i+1}-\sum_{m=N-i+2}^{N}C_{m}(\bar{b_{N-i+1}⊕b_{m}})\right]$$

where index *i* is from 1 to *N* = 12, *C*_T_ is the total capacitance of each DAC branch, and *b*_m_ is the binary bit value. The switching energy *E*_MCS_(i) of the *i*th capacitor in the MCS can be expressed as [15]

(2) $$E_{\mathrm{MCS}}(i)=\left(\frac{1}{2}-\frac{C_{N-i+1}}{2C_{T}}\right)V_{\mathrm{REF}}^{2}C_{N-i+1}+\frac{1}{2}\frac{C_{N-i+1}}{C_{T}}V_{\mathrm{REF}}^{2}\sum_{m=N-i+2}^{N}{\left(-1\right)}^{\bar{b_{N-i+1}⊕b_{m}}}C_{m}$$

A detailed derivation of Equations (1) and (2) can be found in the Appendix A and Appendix B, respectively. In the subranging ADC, switching energy can be divided into MSB and LSB segments of the DAC. The switching energy of the MSB segment can be expressed as

(3) $$E_{\mathrm{DAS}}\left(\mathrm{MSB}\right)=\frac{V_{\mathrm{REF}}^{2}}{2}C_{\mathrm{SW}}\left(1-\frac{C_{\mathrm{SW}}}{C_{T}}\right)$$

where *C*_SW_ is the sum of switched capacitors. The switching energy of the LSB segment is calculated using 6-bit MCS. The total switching energy is obtained using

(4) $$E_{\mathrm{total}}=E_{\mathrm{DAS}}\left(\mathrm{MSB}\right)+\sum_{i=1}^{6}E_{\mathrm{MCS}}\left(i\right)$$

**Figure 5:**
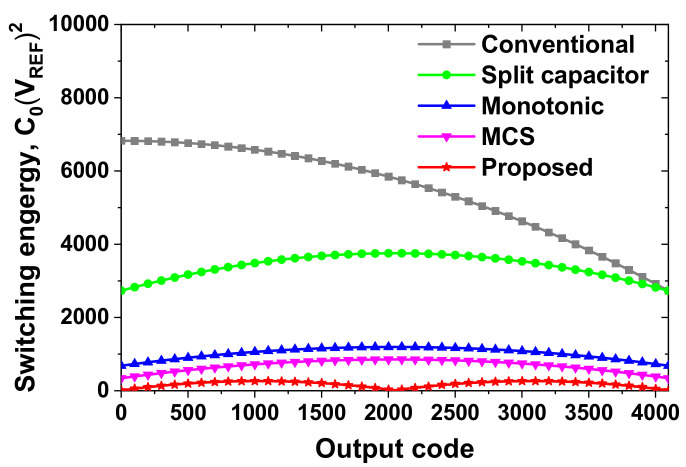
Comparison of the DAC switching energy.

Figure 5 compares the switching energy of a 12-bit ADC normalized using *V*_REF_ and the unit capacitor *C*_1_. The split capacitor scheme saves 37.5% of energy on average compared with the conventional method [1]. The monotonic switching saves up to 81%. The energy is further reduced using the MCS to 87.5%. Finally, the average switching energy saved is up to 96.7% when combining MCS and DAS in the subranging ADC, which is 9.2% lower than the previous state-of-the-art [7]. This result neglects the energy of the 7-bit coarse ADC, which is relatively small compared to the energy of the 12-bit fine ADC. We note that the switching energy is a normalized value using *V*_REF_ and *C*_1_, independent of the technology node. Relatively low power can still be achieved using the conventional method—for example, 0.084 μW for a 10-bit ADC [8] and 0.38 μW for a 12-bit ADC [16]. Because the SAR ADC is realized using mostly digital logic, except for the comparator, low power can be achieved using scaled-down CMOS technology; the works [8] and [16] are realized using 40 nm and 65 nm CMOS processes, respectively.

## 3. Mismatch Calibration

### 3.1. DAC Capacitor Mismatch Calibration

**Figure 6:**
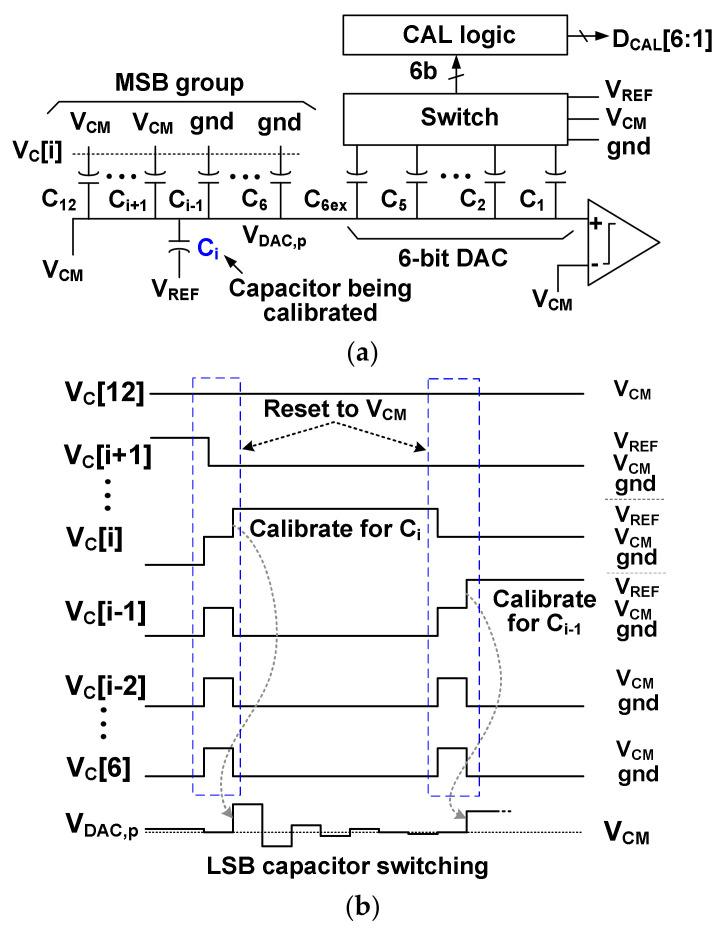
(**a**) Example DAC configuration for reading out the mismatch of the capacitor *C*_i_. (**b**) Waveforms of the control signal *V*_C_[i] at the bottom plate of the DAC capacitor during calibration *C*_i_.

Figure 6a shows one example of a DAC configuration for reading out the mismatch of *C*_i_ (*i* = 6 to 12), one of the 7-bit MSB segments of the DAC. The proposed calibration method reuses the 6-bit DAC to measure the weight error of *C*_i_. The 6-bit DAC consists of 5-bit LSB capacitors (*C*_1_ to *C*_5_) and one extra capacitor *C*_6ex_. Assuming that the 6-bit DAC has sufficient intrinsic linearity, the mismatch of each *C*_i_ is sequentially measured. The digital representation *D*_CAL_[6:1] of the mismatch is generated from the CAL logic. The positive DAC branch is evaluated first, and the negative DAC branch is calibrated next. During the positive DAC calibration, *V*_DAC,n_ (negative input of the comparator) is connected to *V*_CM_.

Figure 6b shows the waveform of *V*_C_[i] during calibration *C*_i_ in the positive branch. Here, *V*_C_[i] is the control signal connected to the bottom plate of the DAC capacitor. In the sampling phase, *V*_C_[i] of all capacitors are connected to *V*_CM_. In the next cycle, the bottom plate of the upper group capacitors (*C*_12_ to *C*_i + 1_) is connected to *V*_CM_, while the bottom plate of the lower group capacitors (*C*_i−1_ to *C*_6_) is connected to the ground. The switching results in *V*_DAC,p_ are

(5) $$V_{\mathrm{DAC},p}=V_{\mathrm{CM}}+(w_{i}^{*}-\sum_{j=6}^{i-1}w_{j}^{*})V_{\mathrm{CM}}$$

where $w_{i}^{*}$ is the weight of *C*_i_ with mismatch error. Without mismatch, *V*_DAC,p_ will be equal to *V*_CM_. The mismatch causes *V*_DAC,p_ to deviate from *V*_CM_, which is measured by the 6-bit DAC. The bit weight difference between *C*_i_ and the sum of lower group capacitors (*C*_i-1_ to *C*_6_), which is quantized by the 6-bit DAC, can be expressed as

(6) $$w_{i}^{*}-\sum_{j=6}^{i-1}w_{j}^{*}=\sum_{j=1}^{6}w_{j}^{}b_{j}+q_{j}$$

where *w*_j_ is the ideal weight, *b*_j_ is the binary value, and *q*_j_ is the quantization error. The values of the LSB segment capacitors (*C*_6ex_, *C*_5_, …, *C*_1_) are assumed to be linear with $w_{j}^{*}=w_{j}$ (*j* = 1, …, 6).

The *C*_6ex_ is added to provide sufficient coverage for weight extraction. The value of *C*_6ex_ is 16*C*_U_, which is small compared to the total capacitance *C*_T_ = 2048*C*_U_ of each DAC, where *C*_U_ = *C*_1_ is the unit capacitor of the DAC. To simplify the SAR logic, *C*_6ex_ can be activated only during the calibration mode while connected to *V*_CM_ in the conversion mode; however, the addition of *C*_6ex_ causes the actual weight of each capacitor to deviate from the ideal binary weight. The DAC mismatch calibration is based on the idea that the addition of *C*_6ex_ does not significantly change the weight of each capacitor. To preserve the correct weight of each capacitor, we handle the issue using an alternative approach: (1) *C*_6ex_ is used in both calibration and conversion mode; in the conversion mode, *C*_6ex_ serves as a redundant capacitor to improve the ADC linearity; (2) mismatch calibration is designed by including the weight of *C*_6ex_; then, the total weight of the 12-bit DAC is increased from 2048 to 2064 (see Table 1).

### 3.2. Mismatch Error of DAC Capacitor

The *V*_DAC,p_ and *V*_DAC,n_ at the inputs of the comparator can be expressed as

(7) $$V_{\mathrm{DAC},p}=V_{\mathrm{IN},p}+\sum_{i\in \Omega }^{}\left(1-2b_{i})(w_{i}-\Delta w_{\mathrm{pi}}\right)V_{\mathrm{CM}}+\sum_{j=1}^{6}(1-2b_{j})w_{j}V_{\mathrm{CM}}$$

(8) $$V_{\mathrm{DAC},n}=V_{\mathrm{IN},n}-\sum_{i\in \Omega }^{}\left(1-2b_{i})(w_{i}-\Delta w_{\mathrm{ni}}\right)V_{\mathrm{CM}}-\sum_{j=1}^{6}(1-2b_{j})w_{j}V_{\mathrm{CM}}$$

where *V*_IN,p_ and *V*_IN,n_ are the sampled input voltages at the positive and negative DAC, respectively. The *b*_i_ is the binary value of the DAC capacitor in the MSB segment (*C*_12_, …, *C*_6_), and *b*_j_ is the value of capacitors in the LSB segment (*C*_6ex_, *C*_5_, …, *C*_1_). The Ω is the group of switched capacitors by the DAS controller. The *w*_i_ is the ideal weight of *i*th capacitor in the MSB segment of the DAC, which is the ratio between *C*_i_ and *C*_T_. The *w*_j_ is the ideal weight of the *j*th capacitor in the LSB segment. The Δ*w*_pi_ and Δ*w*_ni_ are the weight errors of the *i*th capacitor in the positive and negative branches of the fine DAC, respectively. Table 1 shows the ideal weight of each capacitor. The second term of (7) and (8) is the amount of change caused by the mismatch of the MSB capacitors. The third term represents the change caused by the mismatch of the LSB capacitors. At the end of conversion, both *V*_DAC,p_ and *V*_DAC,n_ approach *V*_CM_ as

(9) $$\left(V_{\mathrm{IN},p}-V_{\mathrm{IN},n}\right)+\sum_{i\in \Omega }\left(1-2b_{i}\right)\left(2w_{i}-\Delta w_{\mathrm{pi}}-\Delta w_{\mathrm{ni}}\right)V_{\mathrm{CM}}+2\sum_{j=1}^{6}\left(1-2b_{j}\right)w_{j}V_{\mathrm{CM}}≅0$$

Noting *V*_REF_ = (*V*_IN,p_ + *V*_IN,n_), we can rearrange (9) as

(10) $$2\frac{V_{\mathrm{IN},p}}{V_{\mathrm{REF}}}=\sum_{i\in \Omega }\left(2b_{i}-1\right)\left(w_{i}-\frac{\Delta w_{\mathrm{pi}}+\Delta w_{\mathrm{ni}}}{2}\right)+2\sum_{j=1}^{6}b_{j}w_{j}+\left(-\sum_{j=1}^{6}w_{j}+1\right)$$

By multiplying 2^11^ on both sides of (10), we obtain

(11) $$2^{12}\frac{V_{\mathrm{IN},p}}{V_{\mathrm{REF}}}=\frac{1}{2}\sum_{i\in \Omega }\left(2b_{i}-1\right)\left(W_{i}-\Delta W_{i}\right)+\sum_{j=1}^{6}b_{j}W_{j}+W_{0}$$

where *W*_i_ = 2^12^*w*_i_, Δ*W*_i_ = (Δ*W*_pi_ + Δ*W*_ni_)/2 is the average error of the positive and negative branch, Δ*W*_pi_ = 2^12^(Δ*w*_pi_), *W*_ni_ = 2^12^(Δ*w*_ni_), and *W*_0_ = 2^11^(127/129) = 2016.248. At this moment, the weight error Δ*W*_i_ is unknown, and the method of calculating Δ*W*_i_ is presented in the next subsection.

### 3.3. Weight Error Extraction

We assume that the overall mismatch of the DAC is averaged out and normalize the full scale to one [17]. Then, the sum of weight for *C*_12_ can be expressed as

(12) $$w_{12}^{*}+\sum_{i=6}^{11}w_{i}^{*}+\sum_{j=1}^{6}w_{j}=1$$

The calibration code *d*_12_ for *C*_12_ can be expressed as

(13) $$d_{12}=w_{12}^{*}-\sum_{i=6}^{11}w_{i}^{*}$$

When we substitute (13) into (12), we obtain

(14) $$\frac{1}{2}-w_{12}^{*}=\frac{1}{2}\left(\sum_{j=1}^{6}w_{j}-d_{12}\right)$$

where *w*_j_ is the weight of the capacitors in the 6-bit DAC. We note that $\Delta_{12}=(w_{12}-w_{12}^{*})$Δ_12_ = (*w*_12_ – w^*^_12_) is the weight error of *C*_12_ and *w*_12_ = (64/129) is the ideal weight of *C*_12_. Then, we obtain

(15) $$\Delta_{12}=\frac{1}{2}\left(\sum_{j=1}^{6}w_{j}-d_{12}\right)-\frac{1}{258}$$

Similarly, the sum of weight for *C*_11_ can be expressed as

(16) $$w_{11}^{*}+\sum_{i=6}^{10}w_{i}^{*}+\sum_{j=1}^{6}w_{j}=1-w_{12}^{*}$$

Using the calibration code *d*_11_ for *C*_11_, we obtain

(17) $$d_{11}=w_{11}^{*}-\sum_{i=6}^{11}w_{i}^{*}$$

Noting that Δ_11_ = (*w*_11_ – w^*^_11_) $\Delta_{11}=(w_{11}-w_{11}^{*})$ is the weight error of *C*_11_, where *w*_11_ = (32/129) is the ideal weight, we obtain

(18) $$\Delta_{11}=\frac{1}{2}\left(\sum_{j=1}^{6}w_{j}-d_{11}-\frac{1}{129}-\Delta_{12}\right)$$

Similarly, we obtain the remaining weights. For example, the weight error Δ_6_ of *C*_6_ can be expressed as

(19) $$\Delta_{6}=\frac{1}{2}\left(\sum_{j=1}^{6}w_{j}-d_{6}-\frac{1}{129}-\Delta_{12}-\Delta_{11}-\Delta_{10}-\Delta_{9}-\Delta_{8}-\Delta_{7}\right)$$

The digital representation of sampled input *V*_IN,p_ can be expressed as *D*_IN,p_ = 2^12^(*V*_IN,p_/*V*_REF_). Then, the result (10) can be rearranged as

(20) $$D_{\mathrm{IN},p}=\frac{1}{2}\sum_{i\in \Omega }\left(2b_{i}-1\right)W_{i}-\frac{1}{2}\sum_{i\in \Omega }\left(2b_{i}-1\right)\Delta W_{i}+\sum_{j=1}^{6}b_{j}W_{j}+W_{0}$$

**Figure 7:**
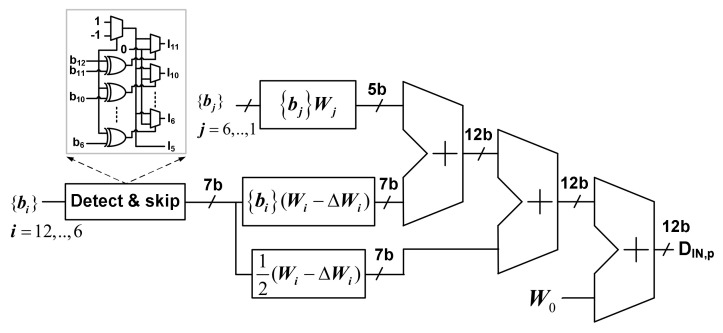
Block diagram of processing of the calibrated digital output.

The first two terms of (20) represent the contribution of the MSB segment of the DAC, which can be positive or negative. The third term is the contribution of the LSB segment. The last term is the average output value. A similar definition can be proposed for *D*_IN,n_ = 2^12^(*V*_IN,n_/*V*_REF_) for the sampled input *V*_IN,n_. Figure 7 shows the block diagram implementing Equation (20) to calculate *D*_IN,p_. Using the logic value of the skipped MSB group, it calculates the contribution of the MSB and LSB segment capacitors. The calculation is performed off-chip using Matlab.

**Figure 8:**
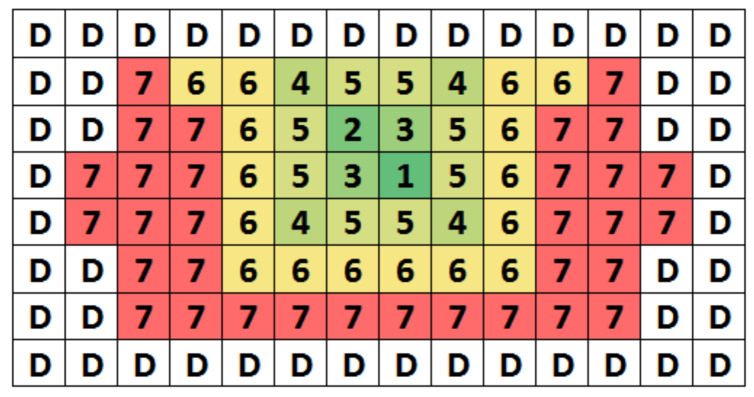
Floor plan of the coarse DAC.

Figure 8 shows the floor plan of the coarse DAC. We use a common-centroid layout to reduce the capacitor mismatch. Because capacitors need to be connected to the outside of the DAC, the metal route increases the coupling with neighboring capacitors. The effect of additional coupling is usually more sensitive to small capacitors. We reduce the effect by placing the capacitors of the LSB segment close to the edge of the DAC. Dummy capacitors are added around the DAC periphery to reduce the mismatch caused by the edge effect. A similar technique is used for the fine DAC.

**Figure 9:**
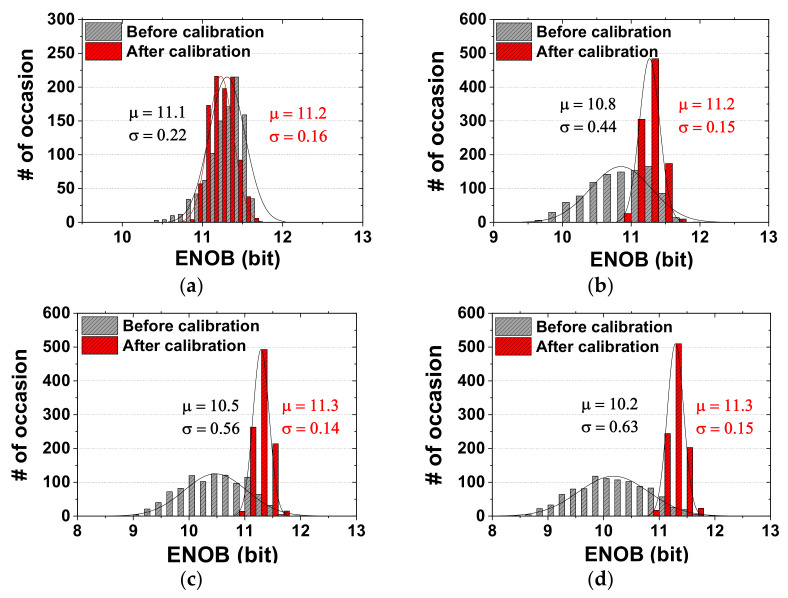
Comparison of the ENOB probability distribution before and after calibration. Mismatch rate is (**a**) 0.5%, (**b**) 1.0%, (**c**) 1.5%, (**d**) 2.0%.

**Figure 10:**
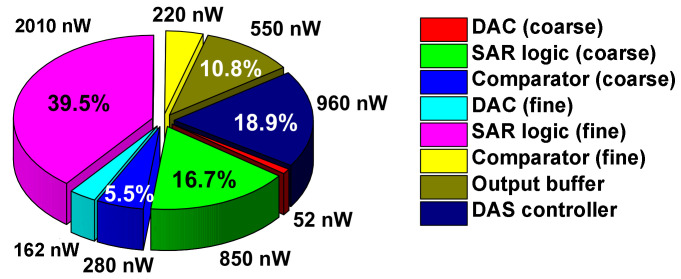
Power breakdown of the ADC.

We use a behavioral model to investigate the ADC performance depending on the mismatch. Monte Carlo simulations with 1000 samples are performed using the DAC capacitor mismatch rate of 1.0%, 1.5%, 2.0%, and 2.5%. Figure 9 compares the effective number of bits (ENOB) probability distribution before and after calibration. Before calibration, the average ENOB decreases from 11.1 bits to 10.2 bits when the mismatch increases from 0.5% to 2%. In the case of a 1% mismatch, the average ENOB increases from 10.8 bits to 11.2 bits after calibration. The standard deviation is reduced from 0.44 bit to 0.15 bit. In the case of a 1.5% mismatch, the average ENOB improves from 10.5 bits to 11.3 bits. The result shows that calibration effectively handles ENOB degradation with the mismatch rate. The minimum capacitor value allowed by the process is 21.2 fF (4 × 4 μm^2^). Based on the process datasheet, the unit capacitor in the coarse DAC is designed to be larger than the minimum value to achieve a 1% mismatch rate, which is 54 fF (6.72 × 6.72 μm^2^). Figure 10 shows the power breakdown of the ADC. Overall power including output buffer is 5.08 μW at *f*_S_ = 200 kS/s. The breakdown shows that the SAR logic of fine ADC, the DAS controller, and the SAR logic of coarse ADC consume 39.5%, 18.9%, and 16.7% of the overall power, respectively.

## 4. Measured Results

**Figure 11:**
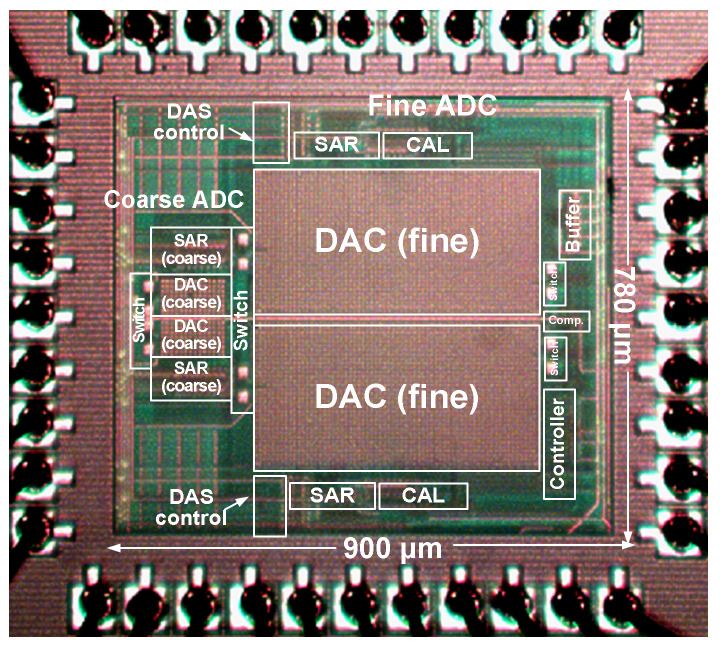
Microphotograph of the fabricated ADC.

Figure 11 shows a microphotograph of the ADC fabricated in a 0.18 μm CMOS process. The core area is 0.7 mm^2^. The coarse ADC occupies 8.5% of the overall area. The IC is mounted on a test board using the chip-on-board (COB) technique. Biopotentials typically exhibit signal frequencies less than 1 kHz. In this measurement, we choose an input frequency *f*_IN_ = 1.12k kHz.

**Figure 12:**
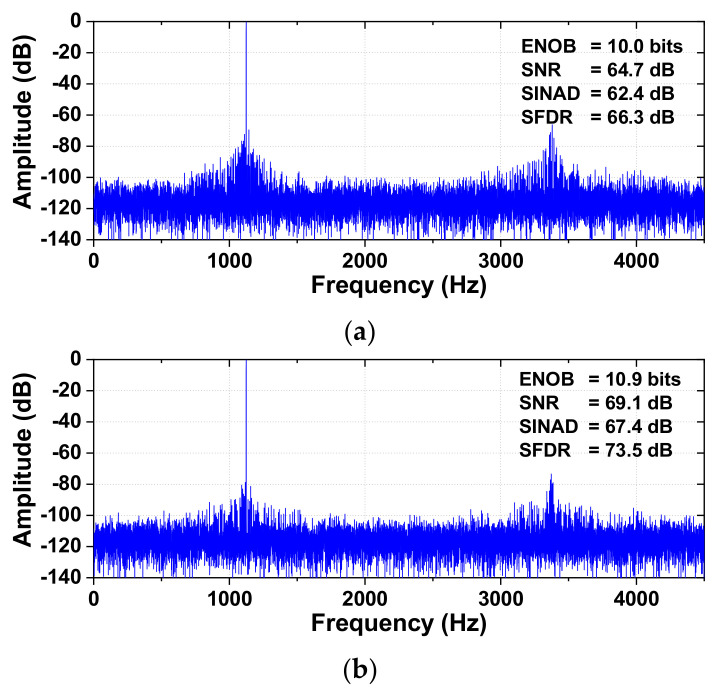
Measured output spectra of the ADC (**a**) before calibration, (**b**) after calibration. *f*_IN_ = 1.124 kHz, *f*_S_ = 9 kS/s.

**Figure 13:**
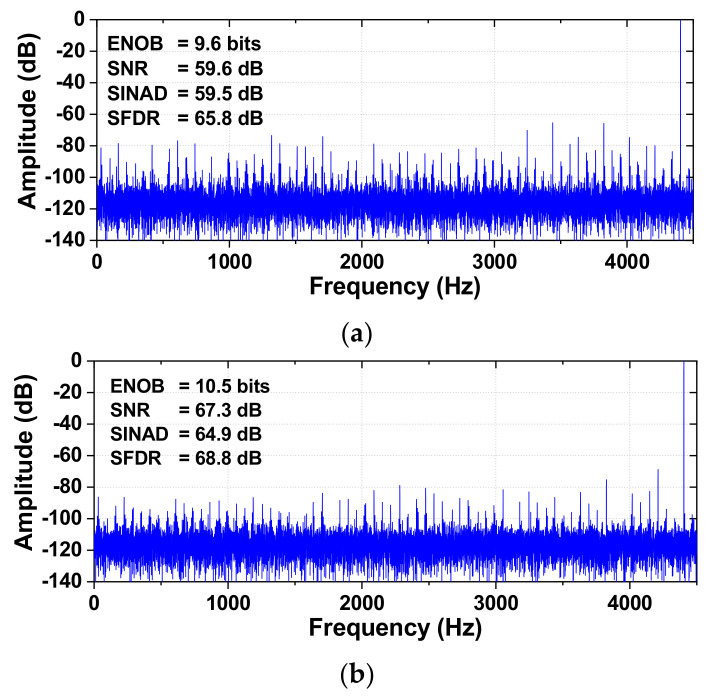
Measured output spectra of the ADC (**a**) before calibration, (**b**) after calibration. *f*_IN_ = 4.403 kHz, *f*_S_ = 9 kS/s.

Figure 12 shows the comparison of the measured output spectra of the ADC before and after calibration. A differential sinusoidal signal with 0.9 V amplitude is applied for dynamic performance testing. The measured data are obtained from the fast Fourier transform (FFT) spectrum with 32768 points. After calibration, SINAD and SFDR are improved by 5.04 dB and 7.21 dB, respectively, resulting in an ENOB of 10.9 bits. The third harmonic located at 3*f*_IN_, which is related to the nonlinearity of the ADC, is reduced from −66.3 dB to −77.2 dB. Figure 13 shows the output spectrum using a near-Nyquist input frequency and the sampling rate *f*_S_ = 9 kS/s. The SINAD and SFDR are improved by 5.44 dB and 2.94 dB, respectively, resulting in an ENOB of 10.5 bits.

**Figure 14:**
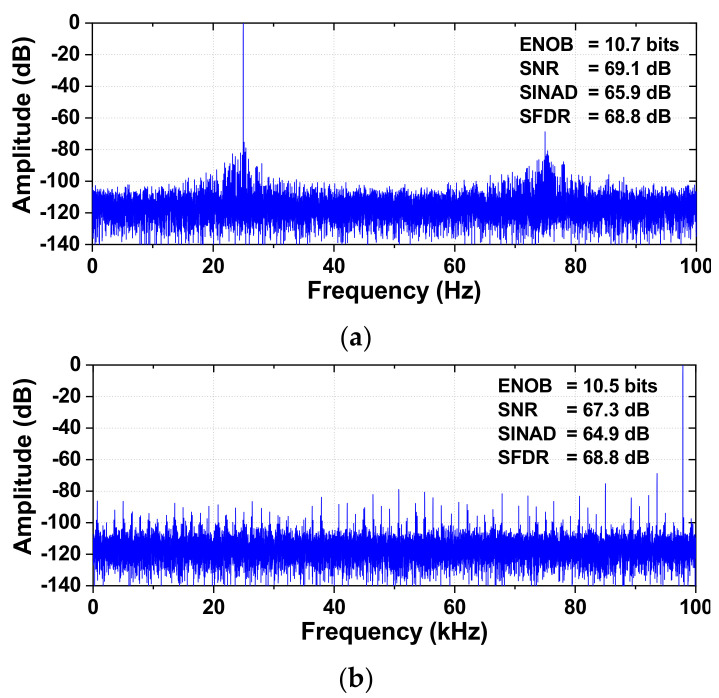
(**a**) Measured output of the ADC. (**b**) Measured output near the Nyquist frequency. *f*_S_ = 200 kS/s.

**Figure 15:**
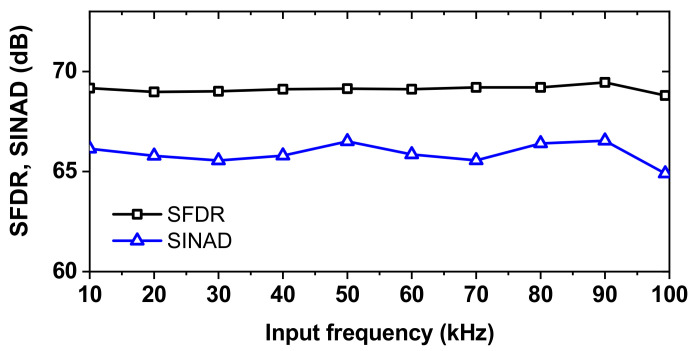
Measured SINAD and SFDR at different input frequencies. *f*_S_ = 200 kS/s.

Additionally, we characterize the dynamic performance at increased *f*_IN_. Figure 14 shows the measured spectra of the ADC at *f*_IN_ = 24.981 kHz and 97.857 kHz (near the Nyquist frequency) after the calibration. The ADC achieves a SINAD of 65.9 dB and an SFDR of 68.8 dB for *f*_IN_ = 24.981 kHz. The level of the third harmonic located at 3*f*_IN_ is −68.7 dB, indicating that further improvement of the ADC nonlinearity is needed. Figure 15 shows the measured SFDR and SINAD as a function of *f*_IN_. The effective resolution bandwidth (ERBW) is the input frequency where the SINAD drops by 3 dB (1/2 LSB or 0.5 bit) from its value for low-frequency input. The result shows that ERBW is around 100 kHz, approximately half of the sampling frequency (Nyquist frequency).

**Figure 16:**
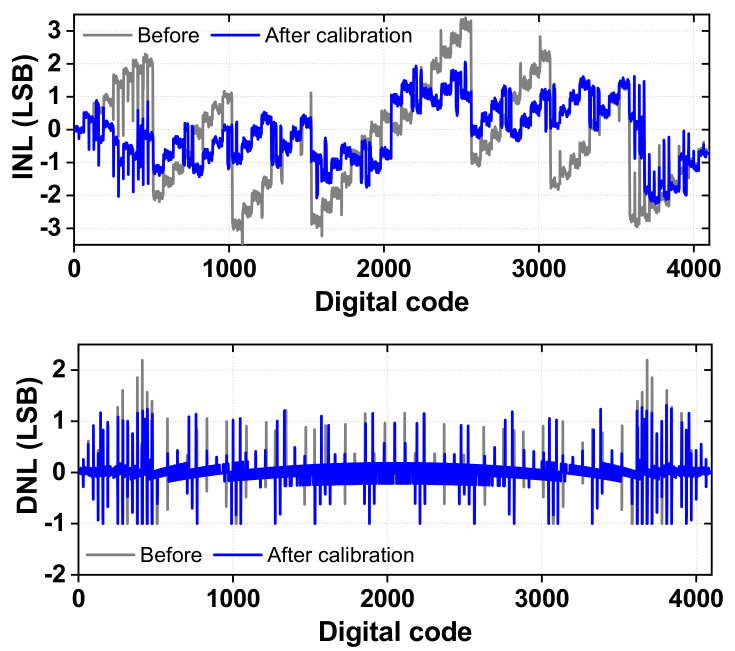
Measured static nonlinearity of the ADC.

Figure 16 compares the differential nonlinearity (DNL) and integral nonlinearity (INL) of the ADC before and after calibration. A total of 32786 codes are collected to build a histogram. Both INL and DNL are improved, and the calibration has a more desirable effect on INL than DNL, as expected. The peak INL decreases from +3.4/−3.48 LSB to +2.05/−2.24 LSB. The peak DNL is +2.19/−1 LSB before calibration, improving to +1.31/−1 LSB.

Table 2 shows the comparison with the previous works. The work in [8] presents a subranging SAR ADC using the DAS method. They use split capacitor switching, which consumes more energy than MCS. A similar observation can be made for the work in [16], which uses the swap-to-reset DAC switching method. The low power consumption can be attributed to the scaled-down technology, 65 nm CMOS [16] and 40 nm CMOS [8,18]. When we compare the ADC realized using a similar CMOS process [2,19], our work achieves a better Walden’s figure-of-merit (FOM_W_) of 13.2 fJ/conv.-step and Schreier’s figure-of-merit (FOM_S_) of 170.4 dB. The work in [11] presents a 13-bit SAR ADC with on-chip calibration realized in a relatively large area (0.9 mm^2^) using a 0.13 μm CMOS process. Our work realizes the subranging ADC, consisting of coarse and fine ADC, in 0.7 mm^2^ using a 0.18 μm CMOS process. The work in [20] presents good dynamic performance; however, it consumes relatively high power, leading to an FoM_S_ of 114.5 dB.

The footnote shows the relationship between ENOB and SINAD. This equation does not explicitly consider the process gain related to the FFT. We can estimate the process gain using *G*_FFT_ = 10∙log(*N*_F_/2), where *N*_F_ is the number of points processed in the FFT. Each *n*th FFT bin can be considered as the output from a narrow bandpass filter with a center frequency at (*nf*_S_/*N*_F_). A large number of samples improves the frequency resolution and decreases the amount of noise in the bin’s passband. For an *N*_F_-point FFT, the average value of the noise contained in each frequency bin is reduced by G_FFT_ below the root-mean-square (rms) value of the quantization noise.

## 5. Conclusions

We investigate a 12-bit subranging SAR ADC for low-power biopotential sensing applications. A new DAC switching method is proposed by combining the MCS and DAS methods, successfully embedded in the subranging structure. Analysis of the DAC switching energy shows that the proposed method saves 96.7% of switching energy compared to the conventional method. To handle the DAC mismatch, we implement digital domain calibration without the extra burden of an on-chip calibration circuit. A simple method of extracting the weight error is presented by reusing the 6-bit DAC. The mismatch data are successfully processed in the digital domain to compensate for the nonlinearity caused by the DAC mismatch. The proposed ADC fabricated in 0.18 μm CMOS demonstrates successful operation and performance improvement using the proposed calibration technique. At a sampling rate of 200 kS/s, the ADC achieves SINAD of 65.9 dB and SFDR of 68.8 dB, with an FoM of 13.2 fJ/conversion- step. The contributions of this paper can be summarized as follows: (1) this work proposes an energy-efficient DAC switching method by combining MCS and DAS, (2) the proposed switching method is successfully implemented in a 12-bit subranging ADC, and (3) this work proposes a digital domain calibration using a normalized full-scale weight method. The result will be useful for realizing a low-power ADC for battery-powered, portable biomedical sensing applications.

## Figures and Tables

**Table 1:** Ideal weight of DAC.

DAC Capacitor	Capacitance (C_U_)	Ideal Weight
*C*_12_	1024	64/129
*C*_11_	512	32/129
*C*_10_	256	16/129
*C*_9_	128	8/129
*C*_8_	64	4/129
*C*_7_	32	2/129
*C*_6_	16	1/129
*C*_6ex_	16	1/129
*C*_5_	8	1/258
*C*_4_	4	1/516
*C*_3_	2	1/1032
*C*_2_	1	1/2064
*C*_1_	1	1/2064
Total	2064	1

**Table 2:** Performance comparison.

	[2]	[8]	[11]	[16]	[18]	[19]	[20]	This Work
Tech. (nm)	180	40	130	65	40	180	65	180
Supply (V)	0.75	0.45	0.5	0.8	1.0	1.0	1.2	1.8/1.0
Resolution (bit)	11	10	13	12	13	11	13	12
Rate (kS/s)	10	200	40	40	6400	1000	50,000	200
SINAD (dB)	60.5	55.6	66.3	64.2	64.1	63.4	70.9	67.4
SFDR (dB)	72.0	76.2	71.0	88.2	68.8	76.6	84.6	73.5
ENOB ^†^ (bit)	9.8	8.95	10.7	10.4	10.4	10.3	11.5	10.9
Calibration	No	No	Yes	No	Yes	No	No	Yes
Power (μW)	0.25	0.084	1.47	0.38	46	24	1000	5.08
Area (mm^2^)	0.13	0.007	0.9	0.11	0.07	0.1	0.05	0.7
FoM_W_ * (fJ/conv.-step)	28.8	0.85	21.8	7.1	2.2	19.9	6.9	13.2
FoM_S_ ** (dB)	163.5	176.4	167.6	171.5	172.5	166.6	114.9	170.4

^†^ ENOB = (SINAD − 1.76)/6.02. ${}^{*}\;{\mathrm{FoM}}_{W}=\frac{\mathrm{Power}}{f_{S}\times 2^{\mathrm{ENOB}}}{,}^{}**{\;\mathrm{FoM}}_{S}=\mathrm{SIN}\mathrm{AD}+10\log\left(\frac{f_{S}}{2\times \mathrm{Power}}\right)$.

**Figure A1:**
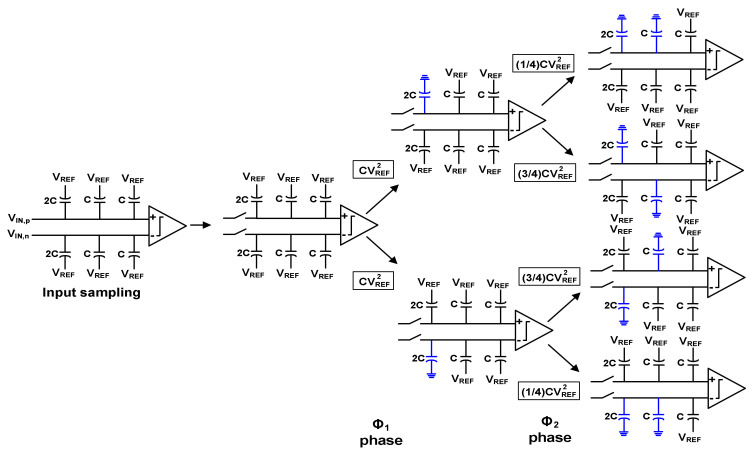
Monotonic switching diagram for a 3-bit ADC.

**Figure A2:**
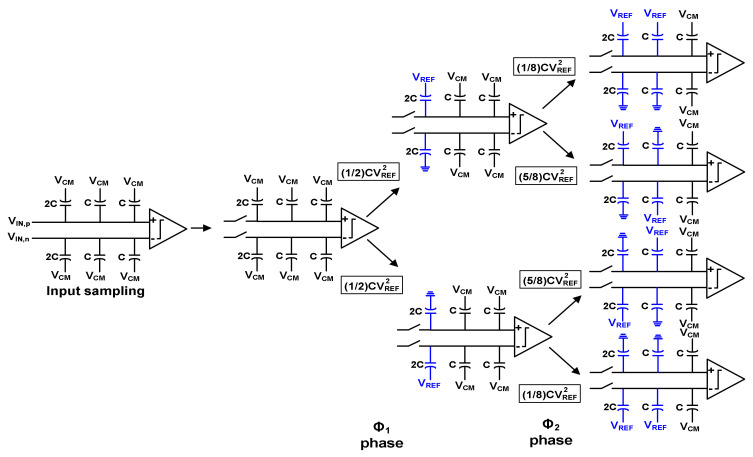
Merged capacitor switching diagram for a 3-bit ADC.

## Appendix A. Monotonic Switching

Figure A1 shows the monotonic switching diagram for a 3-bit example (*N* = 3). After the sampling switches are turned off, the comparator directly performs the first comparison without switching any capacitor.

(1) After the first comparison, the MSB bit *b*_N_ is determined. The bottom plate of capacitor C_N_ (positive DAC branch if *b*_N_ = 1 or negative DAC branch if *b*_N_ = 0) is switched to the ground. Then, the comparator input (*V*_DAC,p_ if *b*_N_ = 1 or *V*_DAC,n_ if *b*_N_ = 0) is reduced by an amount of (*C*_N_/*C*_T_)*V*_REF_. The total capacitance connected to *V*_REF_ is (*C*_T_ − *C*_N_) in the corresponding DAC branch. The switching energy that *V*_REF_ supplies to the DAC at the first cycle (ϕ_1_) can be expressed as

(A1) $$E_{\mathrm{Mono}}(1)=\frac{C_{N}}{C_{T}}V_{\mathrm{REF}}^{2}\left(C_{T}-C_{N}\right)=\frac{1}{2}V_{\mathrm{REF}}^{2}C_{N}=CV_{\mathrm{REF}}^{2}$$

where *C*_N_ = 2*C*, *C*_T_ = 4*C* is the total capacitance of each DAC branch, and *C* is the unit capacitance.

(2) After the second comparison, *b*_N−1_ is determined. The bottom plate of capacitor *C*_N−1_ (positive DAC branch if *b*_N−1_ = 1 or negative DAC branch if *b*_N−1_ = 0) is switched to the ground. Then, the comparator input is reduced by (*C*_N−1_/*C*_T_)*V*_REF_. We consider the four cases as follows:

- If *b*_N_ = 0, *b*_N−1_ = 0: total capacitance connected to *V*_REF_ of the negative DAC branch is *C*_T_ − *C*_N−1_ − *C*_N_;
- If *b*_N_ = 0, *b*_N−1_ = 1: total capacitance connected to *V*_REF_ of the positive DAC branch is *C*_T_ − *C*_N−1_;
- If *b*_N_ = 1, *b*_N−1_ = 0: total capacitance connected to *V*_REF_ of the negative DAC branch is *C*_T_ − *C*_N−1_;
- If *b*_N_ = 1, *b*_N−1_ = 1: total capacitance connected to *V*_REF_ of the positive DAC branch is *C*_T_ − *C*_N−1_ − *C*_N_.

We can express the four cases using a single equation that describes the total capacitance connected to *V*_REF_ as

(A2) $$C_{T}-C_{N-1}-(\bar{b_{N-1}⊕b_{N}})C_{N}$$

where (b_N−1_ ⊕ b_N_) is the XOR of the current bit (b_N_) and the previous bit (b_N−1_) value.

The switching energy that *V*_REF_ supplies to the DAC at the second cycle (ϕ_2_) can be expressed as

(A3) $$E_{\mathrm{Mono}}(2)=\frac{C_{N-1}}{C_{T}}V_{\mathrm{REF}}^{2}\left[C_{T}-C_{N-1}-(\bar{b_{N-1}⊕b_{N}})C_{N}\right]$$

(3) After the third comparison, *b*_N−2_ is determined. The bottom plate of capacitor *C*_N−2_ is switched to the ground. The comparator input is reduced by an amount of (*C*_N−2_/*C*_T_)*V*_REF_. There are eight switching cases, and we can express the cases using a single equation that describes the total capacitance connected to *V*_REF_ as

(A4) $$C_{T}-C_{N-2}-(\bar{b_{N-2}⊕b_{N}})C_{N}-(\bar{b_{N-2}⊕b_{N-1}})C_{N-1}$$

The switching energy that *V*_REF_ supplies to the DAC at the third cycle (ϕ_3_) can be expressed as

(A5) $$\begin{array}{l} E_{\mathrm{Mono}}(3)=\frac{C_{N-2}}{C_{T}}V_{\mathrm{REF}}^{2}\left[C_{T}-C_{N-3+1}-(\bar{b_{N-2}⊕b_{N}})C_{N}-\bar{(b_{N-2}⊕b_{N-1}})C_{N-1}\right]\\ \;\;\;\;=\frac{C_{N-3+1}}{C_{T}}V_{\mathrm{REF}}^{2}\left[C_{T}-C_{N-3+1}-\sum_{m=N-3+2}^{N}C_{m}(\bar{b_{N-3+1}⊕b_{m}})\right] \end{array}$$

By generalizing the above result for an *N*-bit ADC, we obtain Equation (1) of the main text.

## Appendix B. Merged Capacitor Switching

Figure A2 shows the merged capacitor switching diagram for a 3-bit example. After the sampling switches are turned off, the comparator directly performs the first comparison without switching any capacitor. Switching energy calculation is similar to monotonic switching, except that switching is performed from *V*_CM_ rather than *V*_REF_.

(1) After the first comparison, the MSB bit *b*_N_ is determined. The bottom plate of capacitor C_N_ (positive DAC branch if *b*_N_ = 1 or negative DAC branch if *b*_N_ = 0) is switched from *V*_CM_ to *V*_REF_. Then, one comparator input (*V*_DAC,p_ if *b*_N_ = 1 or *V*_DAC,n_ if *b*_N_ = 0) is increased by the amount of (*C*_N_/*C*_T_)(*V*_REF_/2). The opposite comparator input is reduced by the amount of (*C*_N_/*C*_T_)(*V*_REF_/2). The total capacitance connected to *V*_REF_ is *C*_N_. The switching energy that *V*_REF_ supplies to the DAC at the first cycle (ϕ_1_) can be expressed as

(A6) $$E_{\mathrm{MCS}}(1)=\left(\frac{1}{2}-\frac{C_{N}}{2C_{T}}\right)V_{\mathrm{REF}}^{2}C_{N}=\left(\frac{1}{2}-\frac{1}{4}\right)V_{\mathrm{REF}}^{2}2C=\frac{CV_{\mathrm{REF}}^{2}}{2}$$

(2) After the second comparison, *b*_N−1_ is determined. The bottom plate of capacitor *C*_N−1_ (positive DAC branch if *b*_N−1_ = 1 or negative DAC branch if *b*_N−1_ = 0) is switched from *V*_CM_ to *V*_REF_. Then, one comparator input is increased by the amount of (*C*_N−1_/*C*_T_)(*V*_REF_/2). The opposite comparator input is reduced by the amount of (*C*_N−1_/*C*_T_)(*V*_REF_/2). We consider one of the four cases (*b*_N_ = 0, *b*_N−1_ = 0). There are two capacitors (C_N_ and C_N−1_) in the positive branch connected to *V*_REF_. The energy that *V*_REF_ supplies to the DAC at this step can be expressed as

(A7) $$\begin{array}{l} E_{\mathrm{MCS}}(2)=\left(V_{\mathrm{REF}}^{}-V_{\mathrm{CM}}-\frac{C_{N-1}}{C_{T}}\frac{V_{\mathrm{REF}}^{}}{2}\right)C_{N-1}V_{\mathrm{REF}}^{}+\left(V_{\mathrm{REF}}^{}-V_{\mathrm{REF}}-\frac{C_{N-1}}{C_{T}}\frac{V_{\mathrm{REF}}^{}}{2}\right)C_{N}V_{\mathrm{REF}}^{}\\ \;\;\;\;=\left(\frac{1}{2}-\frac{C_{N-1}}{2C_{T}}\right)C_{N-1}V_{\mathrm{REF}}^{2}-\frac{1}{2}\frac{C_{N-1}}{C_{T}}V_{\mathrm{REF}}^{2}C_{N}=\frac{1}{8}CV_{\mathrm{REF}}^{2} \end{array}$$

Similar equations can be derived for the remaining three cases. Then, we can express the four cases using a single equation, and the switching energy at ϕ_2_ can be expressed as

(A8) $$\begin{array}{l} E_{\mathrm{MCS}}(2)=\left(\frac{1}{2}-\frac{C_{N-1}}{2C_{T}}\right)V_{\mathrm{REF}}^{2}C_{N-1}\pm \frac{1}{2}\frac{C_{N-1}}{C_{T}}V_{\mathrm{REF}}^{2}C_{N}\\ \;\;\;\;=\left(\frac{1}{2}-\frac{C_{N-1}}{2C_{T}}\right)V_{\mathrm{REF}}^{2}C_{N-1}+\frac{1}{2}\frac{C_{N-1}}{C_{T}}V_{\mathrm{REF}}^{2}{\left(-1\right)}^{\bar{b_{N-1}⊕b_{N}}}C_{N}=\frac{3}{8}V_{\mathrm{REF}}^{2}C\pm \frac{2}{8}V_{\mathrm{REF}}^{2}C \end{array}$$

where the ± sign is replaced with ${\left(-1\right)}^{\bar{b_{N-1}⊕b_{N}}}$.

(3) After the third comparison, *b*_N−2_ is determined. There are eight cases, and we can express the cases using a single equation. Then, the switching energy at ϕ_3_ can be expressed as

(A9) $$\begin{array}{l} E_{\mathrm{MCS}}(3)=\left(\frac{1}{2}-\frac{C_{N-2}}{2C_{T}}\right)V_{\mathrm{REF}}^{2}C_{N-2}+\frac{1}{2}\frac{C_{N-2}}{C_{T}}V_{\mathrm{REF}}^{2}\left(\pm C_{N}\pm C_{N-1}\right)\\ \;\;\;\;=\left(\frac{1}{2}-\frac{C_{N-2}}{2C_{T}}\right)V_{\mathrm{REF}}^{2}C_{N-2}+\frac{1}{2}\frac{C_{N-2}}{C_{T}}V_{\mathrm{REF}}^{2}\left[{\left(-1\right)}^{\bar{b_{N-2}⊕b_{N}}}C_{N}+{\left(-1\right)}^{\bar{b_{N-2}⊕b_{N-1}}}C_{N-1}\right] \end{array}$$

where the first ± sign in the second term is replaced with ${\left(-1\right)}^{\bar{b_{N-2}⊕b_{N}}}$, and the second ± sign is replaced with ${\left(-1\right)}^{\bar{b_{N-2}⊕b_{N-1}}}$. By generalizing the above result, we obtain Equation (2) of the main text.
